# Durable Control of Mycosis Fungoides after Sepsis: “Coley's Toxin?” Case Report and Review of the Literature

**DOI:** 10.1155/2019/1507014

**Published:** 2019-08-04

**Authors:** Benjamin Heyman, Chris R. Kelsey, Anne Beaven

**Affiliations:** ^1^Division of Regenerative Medicine, Department of Medicine, UC San Diego, Moores Cancer Center, San Diego, CA, USA; ^2^Department of Radiation Oncology, Duke University, Durham, NC, USA; ^3^Division of Hematology/Oncology, Department of Medicine, University of North Carolina, Chapel Hill, NC, USA

## Abstract

Mycosis fungoides, along with Sezary syndrome, is the most common subtype of cutaneous T-cell lymphoma. In this report, we present a patient with advanced-stage mycosis fungoides, who after successful treatment of methicillin-resistant *Staphylococcus aureus* bacteremia had prolonged disease control off systemic therapy. While this may have been due to single-agent gemcitabine, which can result in long remission, we hypothesize that our patient's durable response was in part due to the immune response elicited after treatment of her severe infection.

## 1. Introduction

Cutaneous T-cell lymphomas (CTCL) are a heterogeneous group of extranodal hematologic malignancies with diverse biology and clinical presentations. Mycosis fungoides (MF) is the most common subtype of CTCL, comprising approximately 50% of CTCLs [1]. Sezary syndrome (SS) is the leukemic form of MF, characterized by circulating abnormal *α*/*β* T lymphocytes with immunophenotype of memory cells, but with aberrant expression of common T-cell markers: CD3^dim^, CD4^+^, CD45RO^+^, CCR4^+^, CD2^−^, CD5^−^, CD7^−^, and CD8^−^ [2]. Patients present with persistent and progressive plaques, patches, cutaneous tumors, or erythroderma at isolated or multiple sites [3]. Sezary syndrome consists of an erythrodermic skin presentation in addition to leukemic involvement of MF [4]. Prognosis for early-stage disease MF is excellent, with normal survival for stage Ia. In contrast, patients with stage IV MF at diagnosis have a median OS of 1.5 years [5]. Currently, optimal treatment for MF is controversial as remissions are rare and are usually not durable [6]. We report a case of a patient with advanced-stage MF who after receiving a course of gemcitabine was hospitalized with sepsis and cervical diskitis, treated with antibiotics, and subsequently experienced a durable treatment-free remission of her CTCL after infection clearance.

## 2. Case

**Figure 1:**
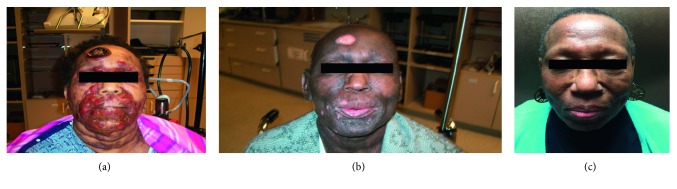
Photograph of cutaneous disease burden at diagnosis (a), after initial radiation therapy (b), and presently (c).

A 61-year-old African American woman with a past medical history of C6-7 spinal stenosis complained of a pruritic rash that began on her face one year prior. The rash appeared raised and erythematous and contained plaques and patches. Despite treatment with topical steroids and emollients, these lesions evolved into cutaneous tumors and spread to encompass >50% of her body surface. The largest tumor was located on her forehead and measured 5 cm (Figure 1(a)). Biopsy of a back lesion demonstrated a pandermal infiltrate of lymphocytes with epidermotropism and pautrier microabscesses in the epidermis. Immunohistochemical stains were positive for CD3, CD4, BF1, CD5, and CD45RA. T-cell clonality studies revealed a monoclonal T-cell receptor (TCR) gene rearrangement for the TCR-*γ* chain. A Sezary preparation demonstrated 73% of abnormal lymphocytes in the blood. Peripheral blood flow cytometry revealed that CD4^+^/CD7^−^ cells comprised 46% of lymphocytes, CD4^+^/CD26^−^ cells comprised 74% of lymphocytes, and the CD4^+^/CD8^+^ ratio was 18 : 1. CT scans demonstrated bilateral axillary and inguinal lymphadenopathy up to 2.9 × 2.5 cm in size, and node biopsy confirmed MF involvement ISCL/EORTC classification N3. Thus, at diagnosis, she had stage IVa (T3N3M0B2) disease.

Initial therapy consisted of interferon-*α* and concomitant total skin electron beam therapy (EBT) (36 Gy). The patient's nodules and tumors on her face, trunk, and back resolved; however, she continued to have significant pruritus and progressive lymphadenopathy (Figure 1(b)). Interferon was poorly tolerated; therefore, the treatment was changed to oral etoposide (50 mg/day). This resulted in temporary stabilization of her systemic disease. However, after four months, she had worsening cutaneous lesions and lymphadenopathy. She was subsequently started on single-agent gemcitabine. Initially, a dose of 1000 mg/m^2^ on days 1, 8, and 15 of a 28-day cycle was planned, but it was decreased to 750 mg/m^2^ every other week starting with cycle 3 due to neutropenia. She completed 13 cycles of gemcitabine with improvement in her cutaneous disease, as she was now found to have lichenification of the arms, abdomen, and lower extremities but continued cracking of hands and feet and she still suffered from pruritus.

**Figure 2:**
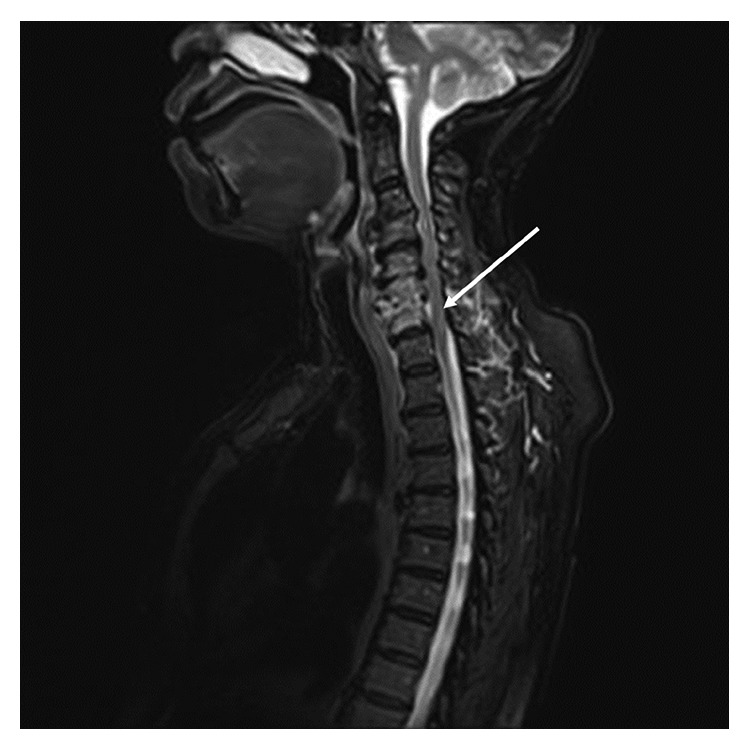
MRI of cervical spine demonstrating epidural abscess from MRSA.

After completing the 13^th^ cycle, the patient presented to the emergency department with fever, hypotension, and new left-sided hemiparesis. She was diagnosed with sepsis, secondary to methicillin-resistant *Staphylococcus aureus* (MRSA) bacteremia. She was not neutropenic. Magnetic resonance imaging (MRI) of her spine revealed T2-hyperintense abnormalities involving the C5–C7 vertebral bodies and the C5-6 and C6-7 disc interspaces, concerning for cord edema/myelomalacia (Figure 2). She subsequently underwent an anterior cervical discectomy and fusion at C6-C7, from which cultures grew MRSA, confirming C6-7 diskitis. After surgery and completion of intravenous antibiotics, she was transitioned to a suppressive antibiotic regimen (rifampin and sulfamethoxazole/trimethoprim). With treatment of the infection and prolonged physical therapy, she regained almost complete use of her left side.

Following discharge, she was seen in follow-up where cutaneous physical exam revealed hyperkeratosis of upper and lower extremities, hypopigmentation of her face without any tumors, and 1 cm bilateral axillary lymphadenopathy. Further systemic treatment of her CTCL was held due to poor performance status, recent severe infection, and low burden of cutaneous disease. The patient was started on narrow-band ultraviolet light therapy for one month, but this was discontinued due to excellent skin symptom control with topical moisturizers. Eighteen months after the MRSA infection, she continued to have very mild cutaneous symptoms off systemic therapy. A Sezary preparation of her peripheral blood revealed 4% abnormal lymphocytes, and peripheral blood flow cytometry revealed CD4^+^/CD7^−^ T cells comprised 2.12% of lymphocytes, CD4^+^/CD26^−^ T cells comprised 1.88% of lymphocytes, and the CD4 : CD8 ratio of the T cells was 1.24 : 1. Restaging computed tomography (CT) scan demonstrated significantly decreased lymphadenopathy, with a left axillary lymph node now measuring 1.2 cm, which decreased from 2.9 cm at diagnosis. There was no evidence of disease progression until 33 months after her infection. At that time, the patient presented with new facial nodules on her left cheek and right forehead, concerning for relapse. Sezary preparation and peripheral blood flow cytometry were still negative for disease. A restaging CT scan revealed stable lymphadenopathy. She received focal RT (4 Gy × 2 to 8 Gy) to her nose, left cheek, and right temple, resulting in complete resolution of her nodules. After the completion of EBT, she has remained off further systemic therapy, which is now 46 months after gemcitabine was stopped for the development of MRSA bacteremia (Figure 1(c)).

## 3. Discussion

We present a patient who was diagnosed with advanced-stage MF and had a durable response off systemic treatment following a prolonged course of gemcitabine followed by MRSA bacteremia and cervical diskitis. The natural history of advanced-stage MF is of relapsing disease with remissions that are rarely durable. For patients with advanced-stage MF, there is no standard of care. Initial treatment for most patients requires both systemic and skin-directed therapies. Initial systemic therapies for advanced-stage MF include interferons, methotrexate, histone deacetylase inhibitors (romidepsin and vorinostat), brentuximab vedotin, psoralen and ultraviolet A, or single-agent cytotoxic chemotherapy [3]. Common chemotherapies include pegylated liposomal doxorubicin, pralatrexate, or gemcitabine [3].

Gemcitabine is a pyrimidine antimetabolite that inhibits DNA synthesis by blocking DNA polymerase and ribonucleotide reductase [7]. Our patient received 13 cycles of gemcitabine because of her aggressive initial presentation, failure to respond to initial therapies, improvement in symptoms, and lack of serious side effects until her infection. Zinzani et al. conducted a phase II clinical trial of single-agent gemcitabine in patients with relapsed or refractory (R/R) CTCLs, of which 30 patients had MF [8]. In patients with MF, the CR rate (CRR) was 10% and the partial response rate (PRR) was 60% [8]. For patients who obtained a CR, responses were ongoing at 6–22 months. Those with partial responses (PR) had a median duration of response ranging from 6 to 10 months [8]. A long-term updated analysis of 39 patients with R/R T-cell lymphoma treated with single-agent gemcitabine demonstrated an ORR of 48% and a CRR of 16%, in the 19 patients who had MF [9]. In the three patients with MF who developed CRs, responses were durable with remissions lasting 10, 18, and 120 months [9]. Lastly, Duvic et al. conducted a phase II clinical trial of single-agent gemcitabine in 33 patients with R/R cutaneous T-cell lymphomas, of which 8 patients were treated off protocol [10]. Thirty-one patients in the study had the diagnosis of MF, with 28 having advanced-stage disease. The ORR for protocol patients was 68%, with a CRR of 12%. The median duration of response for protocol patients achieving a PR was 4.1 months. The estimated median 3-year overall survival was 20.4 months [10]. Thus, single-agent gemcitabine can be effective in some patients, and, although rare, it can produce long-term remissions.

It is possible that our patient's durable response was due to single-agent gemcitabine; however, there is only one reported remission in the literature lasting >2 years. Therefore, we hypothesize that the immune response stimulated by her bacterial sepsis/diskitis may have contributed to the prolonged disease control. This idea was first proposed by William B. Coley in the early 1900s when he demonstrated that the exposure to bacteria/bacterial toxins hindered tumor growth. “Coley's toxins” were heat-killed combinations of *Streptococcus pyogenes* and *Serratia marcescens* that Dr. Coley injected into patients with advanced malignancies [11]. He reported that in 80% of the cases, for which no alternative treatment was available, survival was >5 years. Coley found that fever induction was the best predictor of patient response. The mechanism of action of “Coley's toxins” is not completely understood, but it is thought that the infection-stimulated tumor regression occurred because of an innate immune response, leading to increased expression of tumor necrosis factor (TNF), interferons, and other cytokines [11].

Although “Coley's toxin” never became part of standard care, cancer immunotherapy is a burgeoning area of research and, in the form of interferon alpha, has been a part of MF treatment for many years. Interferon alpha (IFN) was the first cancer immunomodulating agent approved by the FDA, in 1986, for use in hairy cell leukemia. Although not an FDA indication, it is an effective MF treatment with reports of objective responses in over 70% of patients [12, 13].

Since Coley's time, research has moved away from using bacterial vaccines to induce an antitumor response to the development of drugs that more specifically target tumor-immune cell interactions. These immunomodulating drugs target immune system checkpoints such as cytotoxic T lymphocyte antigen 4 (CTLA-4) and programmed cell death protein 1 (PD-1). A phase I trial of the anti-PD-1 monoclonal antibody, nivolumab, reported that in 13 patients with MF, the ORR was 15%, with a median progression-free survival (PFS) of 10 months [14]. A phase II trial of a similar agent, pembrolizumab, reported an ORR of 38% with one CR in 24 patients with MF. The one-year PFS was 69%, suggesting that checkpoint inhibitors may lead to durable responses in MF [15]. Currently, pembrolizumab in combination with radiotherapy is being investigated as a way to enhance the abscopal effect for the treatment of patients with R/R MF (NCT03385226).

## 4. Conclusion

We present data on a patient with advanced MF who had unusually prolonged disease control after single-agent gemcitabine with subsequent MRSA bacteremia and diskitis. Forty-six months after her infection, she has had excellent control of her disease without any systemic therapy since single-agent gemcitabine was stopped. Patients who receive single-agent gemcitabine typically do not achieve such impressive durable control of disease. Therefore, we suspect that her prolonged response was secondary to the severe bacterial infection, resulting in an antitumor immunity similar to Coley's toxin. Currently, there is a lack of reported cases in the literature regarding the role of infection and immune system stimulation on disease outcome in MF. Further reporting and investigation are required to better understand its role and significance in MF.

## References

[1] Criscione V. D., Weinstock M. A. (2007). Incidence of cutaneous T-cell lymphoma in the United States, 1973–2002. *Archives of Dermatology*.

[2] Yawalkar N., Ferenczi K., Jones D. A. (2003). Profound loss of T-cell receptor repertoire complexity in cutaneous T-cell lymphoma. *Blood*.

[3] Foss F. M., Girardi M. (2017). Mycosis fungoides and sezary syndrome. *Hematology/Oncology Clinics of North America*.

[4] Virmani P., Hwang S. H., Hastings J. G. (2017). Systemic therapy for cutaneous T-cell lymphoma: who, when, what, and why?. *Expert Review of Hematology*.

[5] Kim Y. H., Liu H. L., Mraz-Gernhard S., Varghese A., Hoppe R. T. (2003). Long-term outcome of 525 patients with mycosis fungoides and Sézary syndrome: clinical prognostic factors and risk for disease progression. *Archives of Dermatology*.

[6] Quaglino P., Maule M., Prince H. M. (2017). Global patterns of care in advanced stage mycosis fungoides/Sezary syndrome: a multicenter retrospective follow-up study from the cutaneous lymphoma international consortium. *Annals of Oncology*.

[7] Plunkett W., Huang P., Xu Y. Z., Heinemann V., Grunewald R., Gandhi V. (1995). Gemcitabine: metabolism, mechanisms of action, and self-potentiation. *Seminars in Oncology*.

[8] Zinzani P. L., Baliva G., Magagnoli M. (2000). Gemcitabine treatment in pretreated cutaneous T-cell lymphoma: experience in 44 patients. *Journal of Clinical Oncology*.

[9] Zinzani P. L., Venturini F., Stefoni V. (2010). Gemcitabine as single agent in pretreated T-cell lymphoma patients: evaluation of the long-term outcome. *Annals of Oncology*.

[10] Duvic M., Talpur R., Wen S., Kurzrock R., David C. L., Apisarnthanarax N. (2006). Phase II evaluation of gemcitabine monotherapy for cutaneous T-cell lymphoma. *Clinical Lymphoma and Myeloma*.

[11] Hoption Cann S. A., van Netten J. P., van Netten C. (2003). Dr William Coley and tumour regression: a place in history or in the future. *Postgraduate Medical Journal*.

[12] Papa G., Tura S., Mandelli F. (1991). Is interferon alpha in cutaneous T-cell lymphoma a treatment of choice?. *British Journal of Haematology*.

[13] Rupoli S., Goteri G., Pulini S. (2005). Long-term experience with low-dose interferon-alpha and PUVA in the management of early mycosis fungoides. *European Journal of Haematology*.

[14] Lesokhin A. M., Ansell S. M., Armand P. (2016). Nivolumab in patients with relapsed or refractory hematologic malignancy: preliminary results of a phase Ib study. *Journal of Clinical Oncology*.

[15] Khodadoust M., Rook A. H., Porcu P. (2016). Pembrolizumab for treatment of relapsed/refractory mycosis fungoides and sezary syndrome: clinical efficacy in a citn multicenter phase 2 study. *Blood*.

